# Ultrasound pretreatment and fermentation temperature improve phytochemical, antioxidant capacity, and mineral bioaccessibility in tempeh under simulated digestion

**DOI:** 10.1016/j.ultsonch.2026.107738

**Published:** 2026-01-05

**Authors:** Iskandar Azmy Harahap, Joanna Suliburska, Daniela Weber, Tuba Esatbeyoglu

**Affiliations:** a Department of Molecular Food Chemistry and Food Development, Institute of Food and One Health, Gottfried Wilhelm Leibniz University Hannover, Am Kleinen Felde 30, 30167 Hannover, Germany; b Department of Molecular Toxicology, German Institute of Human Nutrition Potsdam-Rehbruecke (DIfE), 14558 Nuthetal, Germany; c Department of Human Nutrition and Dietetics, Faculty of Food Science and Nutrition, Poznan University of Life Sciences, 60-624 Poznan, Poland

**Keywords:** Ultrasound processing, Phytonutrient, Bioaccessibility, Antioxidant capacity, Mineral release

## Abstract

This study investigated how ultrasound pretreatment and controlled fermentation temperature influence phytonutrient release, antioxidant capacity, and mineral bioaccessibility in tempeh under simulated gastrointestinal conditions. Soybeans underwent ultrasound pretreatment soaking and cooking, followed by fermentation with *Rhizopus oligosporus* for 48 h at 30 °C or 36 °C. Total phenolic content (TPC), total flavonoid content (TFC), antioxidant activity (FRAP and CUPRAC), and mineral bioaccessibility (calcium, magnesium, and iron) were measured in both undigested and *in vitro* digested samples. Correlation analyses were conducted to explore functional interactions between phytonutrients, antioxidant potential, and mineral release. Compared with unfermented soybeans, tempeh fermented at 36 °C showed significantly higher TPC, TFC, FRAP, CUPRAC, and calcium concentrations, while magnesium and iron levels remained unchanged. Ultrasound pretreatment soaking and cooking with fermentation at 36 °C promoted TPC and TFC accumulation before digestion and yielded the highest TFC, FRAP, and calcium levels after simulated digestion. Bioaccessibility analysis revealed that TFC, calcium, and iron were significantly higher in ultrasound pretreatment soaking and cooking fermented at 36 °C. A strong positive correlation was observed between flavonoid levels and calcium release (*r* = 0.929, *p* < 0.001). The combination of ultrasound pretreatment and elevated fermentation temperature enhance the release and bioaccessibility of flavonoids, calcium, and iron in tempeh. The strong correlation between flavonoid content and calcium release underscores their interrelated roles. These findings highlight ultrasound pretreatment combined with controlled fermentation temperature as a practical food processing strategy to improve the nutritional profile, bioaccessibility, and functional quality of plant‑based fermented foods under simulated gastrointestinal conditions.

## Highlights

• Fermentation at 36 °C increases phenolics, flavonoids, antioxidants, and calcium.
• Ultrasound pretreatment enhances flavonoid bioaccessibility and calcium release.
• Flavonoid levels correlate strongly with calcium release after in vitro digestion.

## Graphical abstract

**Figure:**
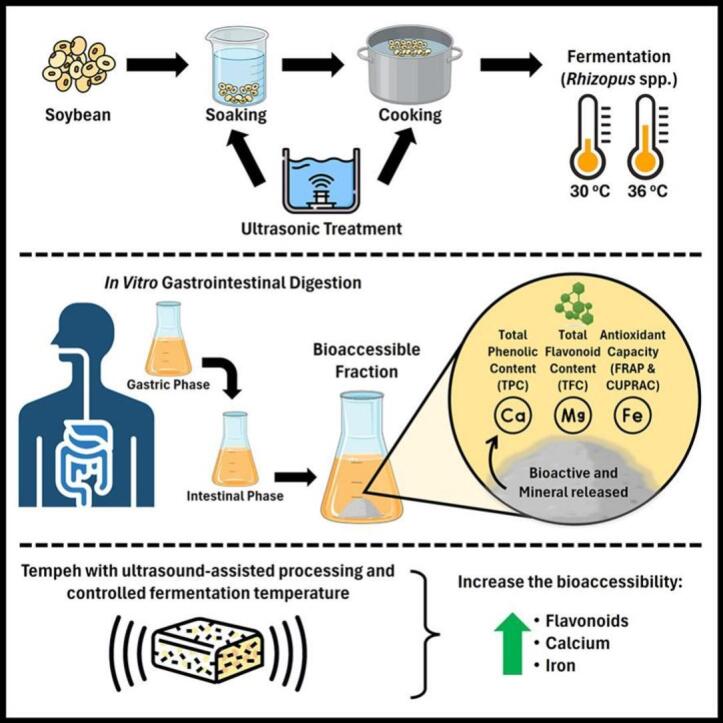

## 1 Introduction

The nutritional quality of plant-based foods has gained increasing scientific and public health interest. Processing conditions play a decisive role in shaping the nutritional functionality and bioaccessibility of phytonutrients and minerals in legumes. Fermentation and emerging nonthermal technologies have shown potential to overcome structural and biochemical barriers that limit the bioavailability of nutraceutical compounds.

Fermented plant-based foods represent an important category of functional foods, with tempeh serving as a notable example. Tempeh has attracted considerable attention as functional foods due to their rich content of bioactive compounds, including polyphenols (e.g., ferulic acid, gallic acid), flavonoids (e.g., quercetin, catechin), and isoflavones (e.g., genistein, daidzein), as well as essential minerals such as calcium, magnesium, and iron [1], [2], [3], [4]. These nutrients play a pivotal role in mitigating age-related oxidative stress, bone loss, and anemia, particularly in postmenopausal women, by supporting cellular redox homeostasis and enhancing endogenous antioxidant defense systems, thereby highlighting the potential of dietary strategies to support healthy aging and alleviate menopause-associated health risks [5], [6]. However, the nutritional efficacy of such foods depends not only on their total content of phytonutrients and minerals but also on their bioaccessibility, which determines the fraction available for absorption during gastrointestinal digestion [7]. The bioavailability of nutraceuticals is influenced by both endogenous factors (gastrointestinal processes) and exogenous factors, including food matrix composition and processing conditions [8]. Plant-based foods face particular challenges due to mineral antinutrients like phytic acid and polyphenols, as well as physical barriers such as cell walls that impair mineral release [9].

Fermentation is a well-established bioprocess known to transform the plant matrix through microbial enzymatic activities. These include the hydrolysis of isoflavone glucosides to more bioactive aglycones, the degradation of antinutrients such as phytic acid, and structural modifications that promote mineral release and solubility. Such biochemical transformations substantially enhance the nutritional and functional properties of legume-based foods, particularly with respect to their contribution to mineral bioaccessibility and antioxidant capacity [1].

In parallel, ultrasound has emerged as a promising physical processing technology in food science. Through cavitation-induced mechanical disruption, ultrasound can break plant cell walls, increase the permeability of cellular structures, and facilitate the release of bound polyphenols and other bioactive constituents. Its application has repeatedly been associated with improved extraction efficiency, enhanced antioxidant potential, and modification of food microstructure in ways that may synergize with fermentation processes [10].

Despite these advancements, most studies have investigated fermentation or ultrasound independently, leaving a critical gap in understanding how their combination influences the bioaccessibility of phytonutrients and minerals under conditions simulating human digestion. Specifically, the extent to which ultrasound pretreatment and fermentation modulates the release of flavonoids, calcium, and iron, and how these interact to affect antioxidant potential and overall nutritional functionality, remains largely unexplored.

To address this knowledge gap, the present study aimed to investigate the combined effects of ultrasound pretreatment combined with controlled fermentation on the release and bioaccessibility of phytoactive compounds, antioxidant capacity, and essential minerals in tempeh during simulated gastrointestinal digestion. Specifically, this study (i) compared two ultrasound pretreatment strategies (during soaking and after cooking), (ii) evaluated fermentation at two controlled temperatures (30 °C and 36 °C), and (iii) examined both undigested and digested matrices to elucidate processing-driven modifications in phytochemical and mineral accessibility. It is hypothesized that ultrasound, by mechanically disrupting the soybean matrix, enhances the release and solubilization of phytonutrients [11], while fermentation temperatures modulate microbial enzymatic activity and generate bioactive metabolites [12], collectively improving the functional and nutritional properties of tempeh. The novelty of this study lies in its dual ultrasound treatment applied during soaking and cooking, its controlled fermentation temperature comparison (30 °C vs. 36 °C), and the assessment of both undigested and digested samples, offering a comprehensive understanding of processing-driven enhancements in nutritional functionality.

By elucidating these effects, this work provides mechanistic insights into how processing strategies can be optimized to design functional fermented foods enriched in bioactives and minerals, with practical implications for improving nutrient bioaccessibility and antioxidant capacity under simulated gastrointestinal conditions. The findings are expected to inform future functional food development and dietary strategies aimed at improving mineral status, antioxidant capacity, and nutrient bioaccessibility under simulated gastrointestinal conditions.

## 2 Materials and methods

### 2.1 Materials

Certified organic, non-GMO yellow soybeans (*Glycine* max) were procured from a local market in Hannover, Germany. Variety and provenance were verified via packaging documentation to ensure batch-to-batch consistency and experimental reproducibility. A commercial tempeh starter culture containing *Rhizopus oligosporus* (Raprima®, Bandung, Indonesia) was employed for all fermentation experiments. This starter is widely used in authentic Indonesian tempeh production and is certified by the Indonesian National Agency for Drug and Food Control (BPOM), guaranteeing both microbial purity and viability. The starter was supplied in powdered form and stored under manufacturer-recommended conditions until use. All chemicals and analytical reagents utilized in this study were of the highest purity grade to ensure reliable biochemical measurements. Ultrapure water was generated using a PURELAB® flex 3 (ELGA LabWater, Veolia Water Technologies, Celle, Germany) and employed for all solution preparations and dilutions. Materials were managed according to manufacturer guidelines, with soybeans and starter cultures stored under conditions preserving their stability and functional integrity.

### 2.2 Tempeh samples preparation

#### 2.2.1 Raw material preparation

Dehulled soybeans were used as the substrates for tempeh fermentation. Three processing schemes were applied to generate distinct pretreatment conditions:

1. Conventional Tempeh:
  Soybeans were soaked and cooked using the conventional method without any ultrasound pretreatment.
2. US (Ultrasound Pretreatment During Soaking):
  Soybeans received a single ultrasound pretreatment prior to soaking. After sonication, the beans were soaked and cooked following the conventional procedure, with no additional ultrasound applied after cooking.
3. USC (Ultrasound Pretreatment During Soaking Followed by Ultrasound Treatment After Cooking):
  This scheme included two ultrasound applications: (i) an ultrasound pretreatment applied before soaking, and (ii) a second ultrasound treatment performed immediately after cooking. The post-cooking ultrasound step was applied exclusively in the USC group because this treatment combination was intended to evaluate potential synergistic effects between pre-soaking and post-cooking ultrasound.

Following each processing scheme, beans were inoculated with *R. oligosporus* and fermented under two controlled temperature conditions (30 °C and 36 °C). This yielded six treatment groups: Tempeh-30, Tempeh-36, US-30, US-36, USC-30, and USC-36.

#### 2.2.2 Ultrasound treatment

In the US scheme, beans were subjected to sonication (Bandelin Sonorex RK 510H, Berlin, Germany; 35 kHz, 120/1480 W, 230 V, 50–60 Hz) for 10 min before overnight soaking at ambient temperature [13], using a solid‑to‑liquid ratio of 1:9 (100 g soybean in 900 mL tap water).

For USC samples, sonication was applied at two points: first before soaking (as in US), and second after thermal cooking (40 min in boiling water), following cooling. Although the ultrasonic bath did not include a built-in temperature control system, the treatment was performed inside a fume hood with a regulated airflow system to dissipate heat and maintain the water temperature within ambient levels (approximately 25–27 °C).

#### 2.2.3 Fermentation process

All pretreated beans, with or without sonication, were cooled at ambient room temperature (approximately 25 °C), inoculated with tempeh starter culture containing *R. oligosporus* (2 g kg^−1^ wet soybean mass), and distributed into sterilized transparent Petri dishes (90 × 15 mm; Sarstedt, Nümbrecht, Germany) at ∼ 2 cm thickness. Fermentation was carried out under strictly regulated incubation at either 30 °C or 36 °C for 48 h in an air-circulating incubator (Memmert® UN55, Memmert, Schwabach, Germany) [14].

#### 2.2.4 Post-fermentation processing

Upon completion, tempeh cakes were immediately freeze-dried to arrest microbial activity and preserve phenolics and minerals. Samples were then finely milled to ensure homogeneity and stored in airtight containers at − 20 °C until analysis.

### 2.3 In vitro gastrointestinal digestion

An *in vitro* gastrointestinal digestion model was employed to evaluate the bioaccessibility of total phenolic content (TPC), total flavonoid content (TFC), antioxidant capacity (FRAP and CUPRAC), and minerals (calcium, magnesium, and iron). The protocol was adapted from established methods for legumes and grain-based matrices [15], [16], [17], with slight modifications.

Briefly, 2 g of powdered sample was dispersed in 20 mL of deionized water and homogenized for 10 min to ensure uniform suspension.

Gastric phase: The pH was adjusted to 2.0 using 0.1 M HCl. A pepsin solution (16 g pepsin per 100 mL 0.1 M HCl) was added at 0.5 mL per 100 mL of homogenate. Samples were incubated at 37 °C for 2 h with gentle stirring, and the pH was maintained using 6 M HCl.

Intestinal phase: The gastric digesta were neutralized to pH 6.8–7.0 using 6 % NaHCO_3_. A pancreatin solution (4 g pancreatin per 1000 mL 0.1 M NaHCO_3_) was added at 10 mL per 40 mL of homogenate, followed by incubation at 37 °C for 2 h. Bile salts were not included, as the study focused on hydrophilic phenolics, antioxidants, and mineral release, for which bile does not play a functional role.

Termination of digestion: Enzymatic activity was stopped by placing the digesta on ice and immediately centrifuging the samples at 4 °C to obtain the bioaccessible fractions.

### 2.4 Post-digestion processing

**Fig. 1:**
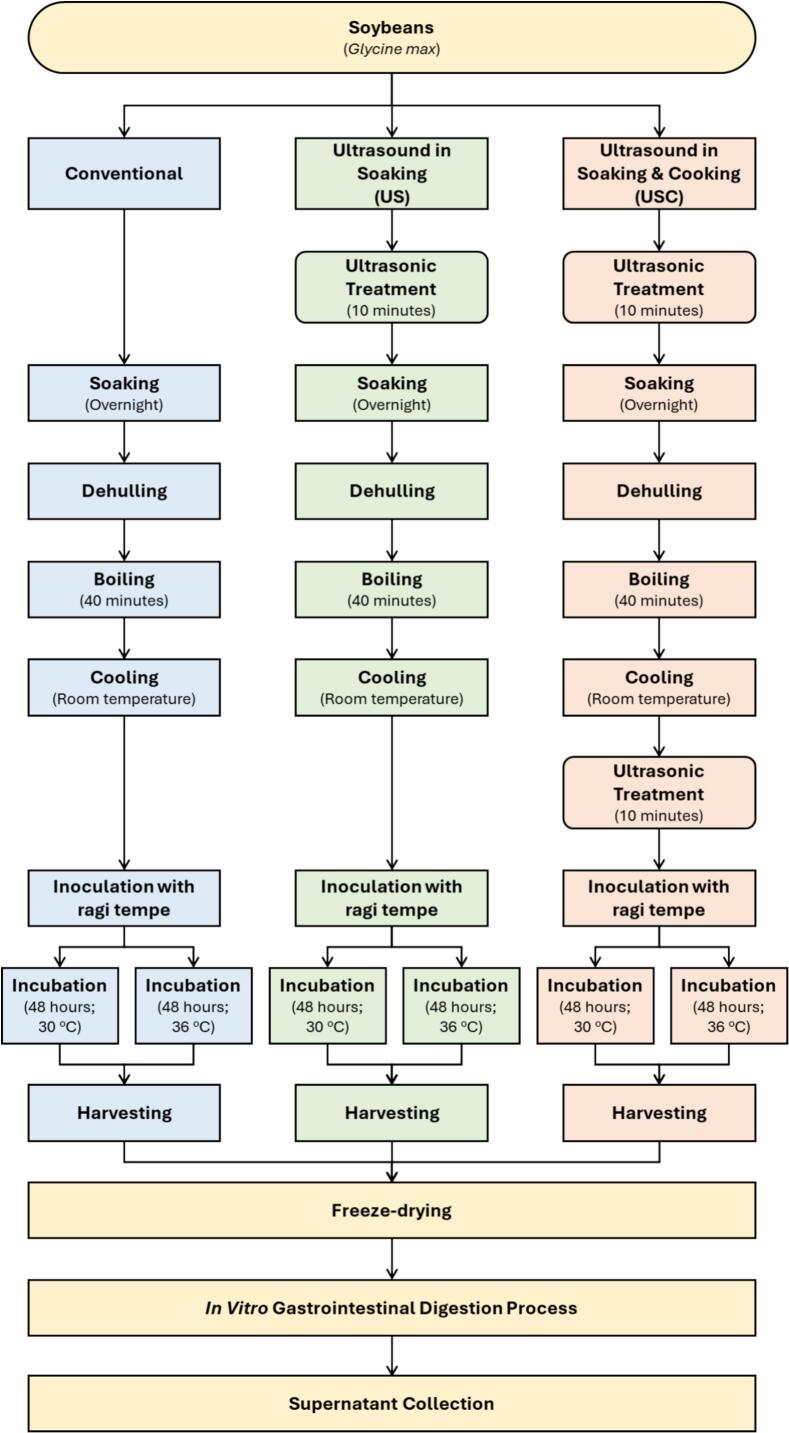
Experimental workflow for tempeh preparation and *in vitro* gastrointestinal digestion.

Upon completion of enzymatic digestion, samples were centrifuged at 3800 rpm for 15 min (Megafuge 8R, Thermo Scientific, Darmstadt, Germany). The supernatants, representing the bioaccessible fraction, were carefully collected and stored at –20 °C until subsequent analysis of phenolics, flavonoids, antioxidant activity, and mineral content. An integrated schematic depicting the overall experimental workflow, from tempeh preparation to simulated gastrointestinal digestion, is provided in Fig. 1.

### 2.5 Phenolics, flavonoids, and antioxidant assays

#### 2.5.1 Sample extraction

Both undigested and digested powders of soybean, Tempeh‑30, Tempeh‑36, US‑30, USC‑30, US‑36, and USC‑36 were used for analysis. For undigested samples, 10 mg of freeze-dried powder was combined with 2 mL of 80 % methanol, vortexed thoroughly, and centrifuged at 14,000 rpm for 10 min at 4 °C (Megafuge 8R, Thermo Scientific, Germany). The supernatants were collected for subsequent measurements. For digested samples, 2 mL aliquots from the simulated gastrointestinal digestion were freeze‑dried to concentrate bioactive constituents and remove excess water. The dried material was then reextracted with 2 mL of 80 % methanol, vortexed, and centrifuged under identical conditions to ensure methodological consistency with undigested samples [18].

#### 2.5.2 Total phenolic content (TPC)

TPC was determined using the Folin–Ciocalteu colorimetric method [19]. Gallic acid served as the reference standard, and 80 % methanol was used as the extraction solvent. The Folin–Ciocalteu reagent (2 N) was diluted to 0.2  N with ultrapure water. A saturated sodium carbonate solution (75 g/L) was prepared for color development. Calibration standards were prepared by serial dilution of gallic acid stock (1 mg/mL) to yield 2.5–100 µg/mL (R^2^ > 0.99). In the assay, 20 µL of sample or standard was mixed with 100 µL of diluted Folin reagent, incubated for 5 min at room temperature, followed by the addition of 100 µL sodium carbonate solution. After 60 min of incubation in the dark, absorbance was measured at 734 nm (Infinite M200 UV–visible spectrophotometer; Tecan, Crailsheim, Germany). Results were expressed as mmol gallic acid equivalents per gram of dry mass (mmol GAE/g d.m.), calculated based on the molar mass of gallic acid (170.12 g/mol).

#### 2.5.3 Total flavonoid content (TFC)

TFC was assessed using an aluminum chloride colorimetric method [20]. Quercetin dihydrate (≥98 %,) was used as the calibration standard. The reagents included 2 % (*w/v*) aluminum chloride hexahydrate in methanol, 50 g/L sodium acetate in distilled water, and methanol. Quercetin stock solution (1 mg/mL) was prepared in methanol and diluted to 500 µg/mL for intermediate calibration; final standards ranged 5–100 µg/mL (R^2^ > 0.99). For analysis, 80 µL of sample or standard was combined with 80 µL AlCl_3_ solution and 120 µL sodium acetate solution in a 96-well microplate. After incubating in the dark at room temperature for 90 min, absorbance was measured at 415 nm. TFC was expressed as mmol quercetin equivalents per gram of dry mass (mmol QE/g d.m.), using quercetin molar mass (302.24 g/mol) for conversion.

#### 2.5.4 Ferric reducing antioxidant power (FRAP)

FRAP assay was conducted using freshly prepared FRAP reagents consisting of sodium acetate buffer (10 mM, pH 3.6), 5 µM TPTZ in 40 mM HCl, and 1 mM FeCl_3_·6H_2_O in a 10:1:1 (*v/v/v*) ratio [21]. Trolox was used as the standard antioxidant. Stock (10 mM) and intermediate (2.5 mM) Trolox solutions were prepared for calibration curves (5–100 µmol/mL, R^2^ > 0.99). For measurement, 50 µL of sample, blank, or standard was added to a 96-well plate with 200  µL FRAP reagent, incubated for 60 min in the dark, and absorbance was read at 593  nm. Results were expressed as mmol Trolox equivalents per gram of dry mass (mmol TE/g d.m.), calculated using the molar mass of Trolox (250.29 g/mol).

#### 2.5.5 Cupric ion reducing antioxidant capacity (CUPRAC)

CUPRAC assays were performed based on the reduction of Cu(II)-neocuproine to Cu(I)-neocuproine chelate [22]. Reagents included Trolox (Sigma-Aldrich, Germany), CuCl_2_ (ChemSolute, Germany), neocuproine (J&K Chemicals, Germany), ammonium acetate buffer (pH 7, ChemSolute, Germany), and 80 % methanol (v/v). Fresh 10 mM CuCl_2_ and 7.5 mM neocuproine solutions were prepared, and Trolox standards (200–4000 μmol/L, R^2^ > 0.99) were generated from a 10 mM stock solution. The reaction mixture contained 5 µL sample or standard, 50 µL each of CuCl_2_, neocuproine, ammonium acetate buffer, and ultrapure water. After 30 min of incubation at room temperature in the dark, absorbance was recorded at 450 nm. Results were expressed as mmol Trolox equivalents per gram of dry mass (mmol TE/g d.m.), using the molar mass of Trolox for conversion.

### 2.6 Mineral analysis

#### 2.6.1 Sample preparation

Freeze-dried undigested and digested powders of soybean, tempeh, and tempeh subjected to ultrasound and different temperature treatments were used for calcium, magnesium, and iron analysis [2], [3], [4]. For digested samples, 2.0 mL aliquots from the *in vitro* gastrointestinal simulation were freeze-dried before analysis to stabilize mineral content. Approximately 1.0 g of each undigested sample was accurately weighed and mineralized using a microwave digestion system (Mars 2™ System; CEM Corporation, USA) by digesting in 65 % (*w/w*) HNO_3_. The resulting digested solution was diluted with deionized water and added with 0.5 % lanthanum(III) chloride (Merck, Darmstadt, Germany) for calcium and magnesium measurements to prevent phosphate interference.

#### 2.6.2 Determination of calcium, magnesium, and iron

Mineral concentrations were determined using a polarized Zeeman atomic absorption spectrophotometer (ZA3000, Hitachi, Tokyo, Japan), with detection wavelengths of 422.7 nm for calcium (Ca) [2], 285.2 nm for magnesium (Mg) [3], and 248.3 nm for iron (Fe) [4]. Method validation was performed using a certified reference material for soybean powder (INCT-SBF-4, Institute of Nuclear Chemistry and Technology, Warsaw, Poland), which provided recovery accuracies of 92 % for Ca, 91 % for Mg, and 97 % for Fe. Analytical reproducibility was confirmed by calculating intra-assay and inter-assay coefficients of variation, which were consistently below 10 % and 12 %, respectively. Results were expressed as micrograms per gram of dry mass (µg/g d.m.).

### 2.7 Bioaccessibility (%) of phytonutrients and minerals

The *in vitro* gastrointestinal digestion model described in Section 2.3 was used to evaluate the bioaccessibility of total phenolic (TPC), total flavonoids (TFC), antioxidant capacity (FRAP and CUPRAC), and minerals (Ca, Mg, Fe) in soybean, tempeh, and tempeh subjected to ultrasound and temperature treatments. Bioaccessibility (%) was calculated to quantify the fraction of each compound released into the soluble phase during simulated digestion relative to its content in the undigested sample.

For each analyte, the following formula was applied:

$$\%Bioaccessibility=\frac{\mathrm{Concentrationindigestedsupernatant}}{\mathrm{Concentrationinundigestedsample}}\times 100$$

where:

• Concentration in digested supernatant corresponds to the amount of analyte (TPC, TFC, FRAP, CUPRAC, Ca, Mg, Fe) measured in the soluble fraction obtained after simulated gastrointestinal digestion.
• Concentration in the undigested sample corresponds to the analyte content measured in the original powdered sample before digestion.

### 2.8 Statistical analysis

All data analyses were conducted using SPSS Statistics for Windows, version 22.0 (IBM Corp., Armonk, NY, USA). Each experimental condition was performed in triplicate (*n* = 3) to ensure reproducibility and to capture experimental variability, which allows for reliable estimation of means and standard deviations (SD). Data are presented as mean ± SD throughout. Before inferential testing, datasets were evaluated for normality using the Shapiro–Wilk test and for homogeneity of variance using Levene’s test. These preliminary checks ensured the appropriateness of parametric analyses. For comparisons among multiple treatments, one-way analysis of variance (ANOVA) was performed. Where significant differences were detected, Tukey’s Honestly Significant Difference (HSD) post hoc test was applied to identify pairwise differences between groups. For direct comparisons between undigested and digested samples (before and after simulated gastrointestinal digestion), a paired Student’s T-test was conducted. Statistical significance was set at *p* < 0.05 for all analyses. Additionally, bar plots with error bars representing SD were generated directly in SPSS to visually illustrate the results. This combination of rigorous statistical testing in tables and graphical representation provides a comprehensive assessment of the effects of ultrasound processing and fermentation temperature on phytonutrient and mineral bioaccessibility.

## 3 Results

### 3.1 Phytochemical, antioxidant, and mineral profiles of soybeans and tempeh at different fermentation temperatures

**Fig. 2:**
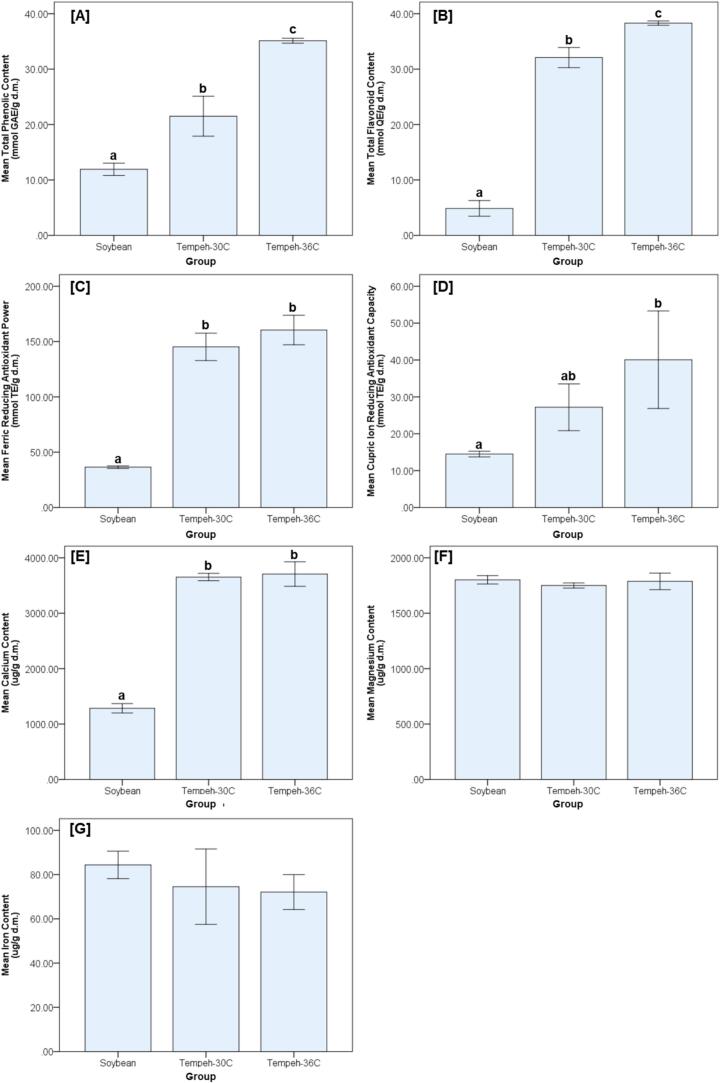
Comparative analysis of phytoactive compounds, antioxidant capacity, and mineral profiles in soybean and tempeh fermented at 30 °C and 36 °C.

Fermentation markedly altered the levels of phytoactive compounds and antioxidant capacity in comparison with unfermented soybeans (Fig. 2). The TPC levels increased significantly following fermentation, with tempeh produced at 30 °C showing almost a 2-fold elevation, while fermentation at 36 °C resulted in an increase of nearly 3-fold compared to soybean. A similar trend was observed for TFC levels, which rose more than 6–fold in tempeh fermented at 30 °C compared to soybean, and reached almost an 8-fold elevation at 36 °C.

Antioxidant activity, as assessed by the FRAP assay, also demonstrated pronounced improvements with fermentation. Tempeh groups exhibited a more than 4-fold increase compared to soybean, although no significant difference was observed between the two fermentation temperatures (30 °C and 36 °C). In contrast, the CUPRAC assay revealed a significant enhancement only at 36 °C, where antioxidant capacity nearly tripled compared to soybean, whereas tempeh produced at 30 °C did not differ significantly from the soybean.

Mineral analysis revealed differential responses to fermentation. Calcium levels were significantly elevated in tempeh compared with soybeans, yet the two fermentation temperatures did not differ from each other. Conversely, magnesium and iron concentrations remained unaffected, with no significant variations detected among soybean and either tempeh group.

Bar graphs illustrate the mean values (± standard deviation) of seven parameters measured across three sample groups: unfermented soybean, tempeh fermented at 30 °C, and tempeh fermented at 36 °C. The parameters include [A] Total Phenolic Content (TPC), [B] Total Flavonoid Content (TFC), [C] Ferric Reducing Antioxidant Power (FRAP), [D] Cupric Ion Reducing Antioxidant Capacity (CUPRAC), and mineral concentrations of [E] calcium, [F] magnesium, and [G] iron. All values are expressed on a dry mass (d.m.) basis. Statistical analysis was performed using one-way ANOVA to assess group differences, followed by Tukey’s Honest Significant Difference (HSD) post hoc test to determine pairwise significance (*p* < 0.05). Homogeneity of variances was confirmed before ANOVA using Levene’s test. Different lowercase letters (a, b, c) above the bars indicate statistically significant differences among groups for each parameter. The absence of superscript letters indicates that no statistically significant differences were observed among the groups. All measurements were conducted in triplicate (*n* = 3).

### 3.2 Effects of ultrasound pretreatment and fermentation temperature on phytochemical, antioxidant, and mineral levels before and after digestion

**Fig. 3:**
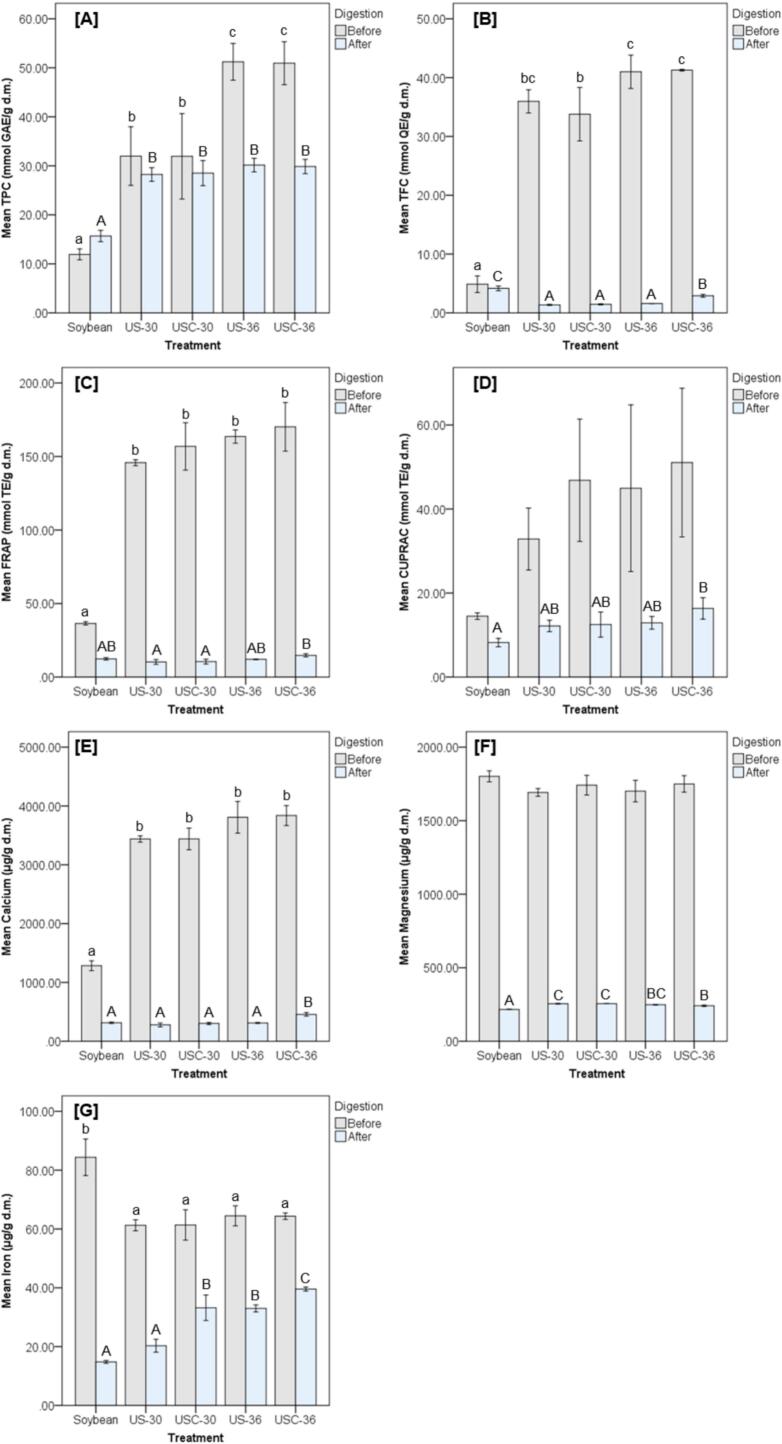
Phytochemical, antioxidant, and mineral levels before and after digestion in soybean and tempeh under simulated gastrointestinal digestion.

Fig. 3 illustrates the combined effects of ultrasound pretreatment and fermentation temperature on phytochemical levels, antioxidant capacity, and mineral release before and after simulated gastrointestinal digestion, using untreated soybean as the reference.

Before digestion, compared to soybean, all ultrasound-treated and fermented groups (US-30, USC-30, US-36, and USC-36) exhibited significantly higher levels of TPC, TFC, and FRAP. Among these, USC-36 demonstrated the greatest increases, showing the highest TPC, TFC, FRAP, and CUPRAC values. Although differences in CUPRAC were not statistically significant, all treated groups displayed higher CUPRAC values than soybean.

For minerals, calcium levels increased significantly in all treated groups compared with soybean, whereas magnesium concentrations did not differ across treatments. In contrast, iron levels decreased significantly in US-30, USC-30, US-36, and USC-36 compared with soybeans.

After digestion, TPC levels remained significantly higher in all treatment groups relative to soybean. Conversely, TFC levels were significantly reduced in US-30, USC-30, US-36, and USC-36 compared with soybean. FRAP values did not differ significantly from soybeans after digestion. However, USC-36 exhibited significantly higher FRAP activity than US-30 and USC-30. For CUPRAC, USC-36 showed a significant increase, surpassing both soybean and the other treatment groups.

Mineral bioaccessibility displayed notable treatment-dependent differences. Calcium bioaccessibility was highest in USC-36, which exceeded soybean and all other groups significantly. Magnesium release was significantly higher in US-30 and USC-30 compared with soybean and with USC-36. For iron, USC-36 again showed the highest post-digestion levels, significantly surpassing soybean and all other treatments.

Bar graphs illustrate the mean values (± standard deviation) of seven analytical outcomes measured across five sample groups: untreated soybean, US-30C (ultrasound-soaked, fermented at 30 °C), USC-30C (ultrasound-soaked and cooked, fermented at 30 °C), US-36C (ultrasound-soaked, fermented at 36 °C), and USC-36C (ultrasound-soaked and cooked, fermented at 36 °C). The assessed outcomes include [A] Total Phenolic Content (TPC), [B] Total Flavonoid Content (TFC), [C] Ferric Reducing Antioxidant Power (FRAP), [D] Cupric Ion Reducing Antioxidant Capacity (CUPRAC), and mineral concentrations of [E] calcium, [F] magnesium, and [G] iron. All values are expressed on a dry mass (d.m.) basis. Statistical analysis was performed using one-way ANOVA to assess group differences, followed by Tukey’s Honest Significant Difference (HSD) post hoc test to determine pairwise significance (*p* < 0.05). For before digestion data, distinct lowercase letters (a, b, c) above the bars indicate statistically significant differences among groups for each analytical outcome. For after digestion data, distinct uppercase letters (A, B, C) denote significant differences. The absence of superscript letters indicates that no statistically significant differences were observed among the groups. All measurements were conducted in triplicate (*n* = 3).

### 3.3 Bioaccessibility of phytochemicals, antioxidant capacity, and minerals under simulated digestion

**Fig. 4:**
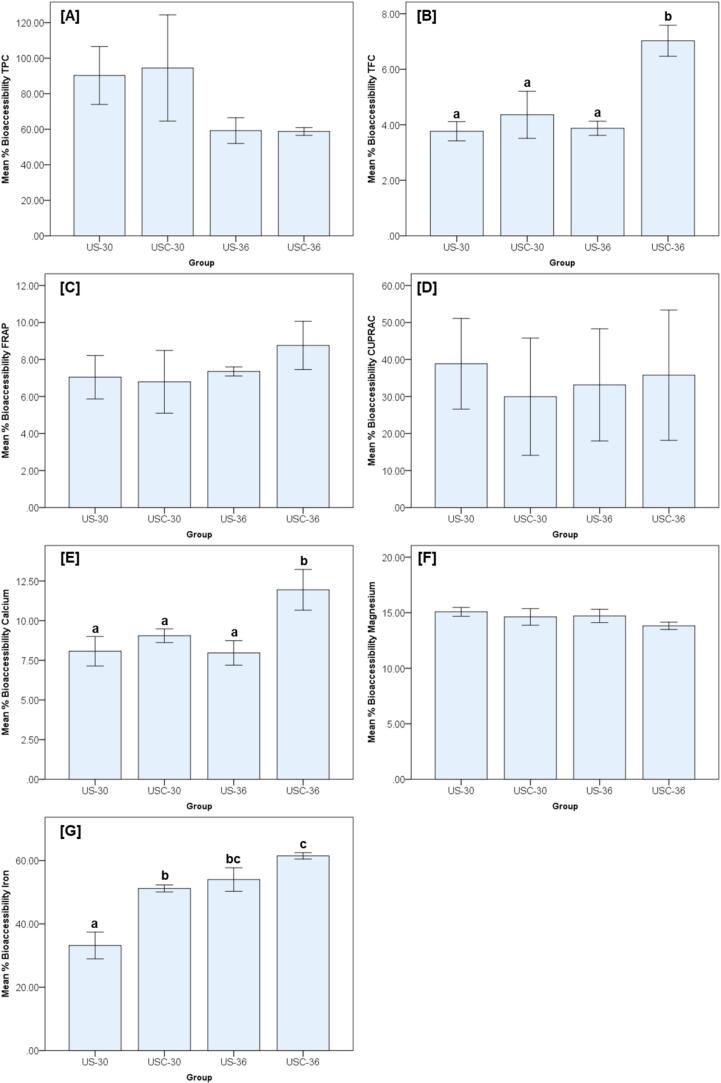
Percentage bioaccessibility of phytochemical compounds, antioxidant capacity, and mineral profiles in tempeh under simulated gastrointestinal digestion.

Fig. 4 demonstrates the bioaccessibility patterns of phytoactive compounds, antioxidant capacity, and minerals following simulated gastrointestinal digestion. No significant differences were observed among the treatment groups for TPC, FRAP, CUPRAC, or magnesium. In contrast, significantly different effects were evident for TFC, calcium, and iron. The bioaccessibility of TFC was significantly enhanced in the USC-36 group, which yielded nearly a twofold increase compared to the other treatments. Calcium followed a similar trend, with USC-36 exhibiting the highest release, representing approximately a 40–50 % increase compared to the other groups. For iron, bioaccessibility was also maximized in USC-36, with values nearly doubling those observed in US-30, while USC-30 and US-36 showed no significant difference.

Bar graphs present the mean percentage bioaccessibility (± standard deviation) of phytoactive compounds, antioxidant capacity, and mineral profiles in tempeh produced under four processing and fermentation conditions: US‑30C (ultrasound pretreatment soaking, fermented at 30 °C), USC‑30C (ultrasound pretreatment soaking and cooking, fermented at 30 °C), US‑36C (ultrasound pretreatment soaking, fermented at 36 °C), and USC‑36C (ultrasound pretreatment soaking and cooking, fermented at 36 °C). The parameters evaluated include [A] Total Phenolic Content (TPC), [B] Total Flavonoid Content (TFC), [C] Ferric Reducing Antioxidant Power (FRAP), [D] Cupric Ion Reducing Antioxidant Capacity (CUPRAC), and mineral concentrations of [E] calcium, [F] magnesium, and [G] iron. All values are expressed on a dry mass (d.m.) basis to ensure comparability across treatments. Percentage bioaccessibility was calculated using the formula: % Bioaccessibility = (Concentration after digestion / Concentration before digestion) × 100. Statistical analysis was performed using one-way analysis of variance (ANOVA) to compare bioaccessibility values among treatments for each parameter, followed by Tukey’s Honest Significant Difference (HSD) post hoc test to determine pairwise differences at *p* < 0.05. Homogeneity of variances was confirmed using Levene’s test before ANOVA, and different lowercase superscript letters (a, b, c) above the bars indicate statistically significant differences among treatments for each parameter. The absence of superscript letters indicates that no statistically significant differences were observed among the groups. All measurements were conducted in triplicate (*n* = 3).

### 3.4 Correlations among phytochemicals, antioxidant capacity, and mineral release after digestion

Pearson correlation analysis was conducted to explore the relationships among phytoactive compounds, antioxidant capacity, and mineral release following simulated gastrointestinal digestion (Table 1). Significant positive correlations were observed between TFC and FRAP (*r* = 0.888, *p* < 0.001) as well as TFC and CUPRAC (*r* = 0.704, *p* = 0.011). For minerals, TFC exhibited a significant negative correlation with magnesium (*r* = –0.786, *p* = 0.002) and a positive correlation with iron (*r* = 0.717, *p* = 0.009). Similarly, antioxidant indices showed significant associations with mineral levels, where FRAP correlated positively with both calcium (*r* = 0.773, *p* = 0.003) and iron (*r* = 0.742, *p* = 0.006), while a significant negative correlation was observed with magnesium (*r* = –0.774, *p* = 0.003). Moreover, the strongest positive correlation detected between TFC and calcium (*r* = 0.929, *p* < 0.001) under simulated gastrointestinal conditions.

**Table 1:** Pearson’s Correlation Coefficients Between Phytochemicals, Antioxidant Capacity, and Mineral Release After Digestion.

**Variables**	**Correlations**	**Coefficient**	**Significance**
Phytochemicals and Antioxidants	TFC – FRAP	0.888	0.000**
	TFC – CUPRAC	0.704	0.011*
	TPC – FRAP	0.501	0.097
	TPC – CUPRAC	−0.217	0.499

Phytochemicals and Minerals	TFC – Magnesium	−0.786	0.002*
	TFC – Iron	0.717	0.009*
	TFC – Calcium	0.929	0.000**
	TPC – Magnesium	−0.363	0.246
	TPC – Iron	0.507	0.092
	TPC − Calcium	0.264	0.406

Antioxidants and Minerals	FRAP – Calcium	0.773	0.003*
	FRAP – Magnesium	−0.774	0.003*
	FRAP – Iron	0.742	0.006*
	CUPRAC – Calcium	0.569	0.054
	CUPRAC – Magnesium	−0.459	0.134
	CUPRAC – Iron	0.409	0.186

The table presents Pearson’s correlation coefficients (*r*) and associated significance levels (*p*-values) describing the relationships among Total Phenolic Content (TPC), Total Flavonoid Content (TFC), Ferric Reducing Antioxidant Power (FRAP), Cupric Ion Reducing Antioxidant Capacity (CUPRAC), and mineral concentrations of calcium, magnesium, and iron in tempeh produced with ultrasound pretreatment processing at different fermentation temperatures following simulated gastrointestinal digestion. Correlation analysis was performed using Pearson’s product–moment correlation to assess the strength and direction of linear associations between variables. Positive coefficients indicate a direct relationship, whereas negative coefficients indicate an inverse relationship. Statistical significance (*) was determined at *p* < 0.05, with *p* < 0.001 considered highly significant (**).

## 4 Discussion

This study evaluates how ultrasound pretreatments (soaking and combined soaking–cooking) and controlled fermentation temperature (30 °C vs. 36 °C) influence the phytochemical composition, antioxidant function, and mineral bioaccessibility of tempeh before and after simulated gastrointestinal digestion. The dual-intervention approach, combining mechanical (ultrasound) and thermal (fermentation temperature) indications, and the assessment of both undigested and digested fractions, provides novel insight into processing-driven changes in nutritional functionality.

Fermentation temperature is a primary driver of polyphenol and flavonoid enrichment. The selected conditions (30 °C and 36 °C for 48 h) reflect temperature and time parameters commonly employed in both traditional and industrial tempeh production. Previous work has confirmed that *R. oligosporus* maintains vigorous growth across a thermal range including 30 °C, 37 °C, and 42 °C [14]. The choice of a 48 h fermentation period was based on evidence indicating that the most pronounced increases in polyphenol concentration and isoflavone aglycone formation occur during the second day of fermentation [23]. Before digestion, samples fermented at 36 °C show substantially higher TPC and TFC compared to 30 °C and raw soybean controls. This pattern is consistent with enhanced microbial metabolism and increased hydrolytic enzyme activity, particularly β-glucosidase and other carbohydrolases, at warmer incubation conditions, which can cleave glycosides and release aglycones and other phenolic compounds. For instance, a prior study demonstrated that solid-state fermentation produced high β-glucosidase activity, converting isoflavone glucosides to aglycones and increasing total phenolic content 1.9-fold [24]. However, the lack of a proportional rise in bulk antioxidant readouts (FRAP, CUPRAC) before digestion suggests that total phenolic mass alone does not determine redox function; instead, the antioxidant response depends on the molecular identity, redox potential, and interaction network of released compounds. Polymeric phenolic compounds demonstrate enhanced antioxidant properties and increased stability compared to their monomeric counterparts [25]. Plant polyphenols encompass diverse structures, including phenylpropanoids, flavonoids, and various oligomeric and polymeric compounds [26]. The redox potential of these compounds creates a complex network where polyphenols can act simultaneously as antioxidants and pro-oxidants, with the catechol group oxidation to o-quinone being particularly important [27]. This redox balance, maintained by oxidase and reductase enzymes, generates reducing species that activate the NRF2/ARE axis, regulating cellular antioxidant responses and demonstrating how molecular identity and interaction networks determine antioxidant efficacy [27], [28].

Ultrasound pretreatment—especially when applied during both soaking and cooking—appears to amplify the accessibility of bound phytochemicals and minerals by generating cavitation-induced shear and microjetting that breach cell walls and disrupt protein–polysaccharide matrices. Ultrasound processing enhances phenolic compound bioaccessibility by cleaving bonds between phenolic compounds and matrix macromolecules and damaging microstructural barriers like cell walls [29]. The ultrasound treatment duration in this study was selected based on a previous finding that a 10-minute exposure yielded the highest total polyphenol content and antioxidant activity in soybeans [13].

In mineral processing, ultrasonic mechanisms include transient cavitation, stable cavitation, and acoustic radiation force effects that influence flotation behavior [29]. Ultrasound treatment affects mineral surface wettability through the generation of hydroxyl radicals and surface modifications, creating iron-deficient surfaces on chalcopyrite, OH and H radicals on pyrite, and silanol groups on quartz [30]. In soybeans specifically, optimized ultrasound pretreatment (55 °C, 15 min, 24 W/cm^2^) increased total phenolic content, total aglycone content, and genistein concentration by 95 % [11]. The cavitation phenomenon produces microstreaming, microjetting, and free radical formation, which can modify cellular permeability and structural integrity [31]. This mechanical effect likely increases substrate exposure to *Rhizopus* enzymes during fermentation, thereby linking between physical and biological mechanisms to enhance the extractability of flavonoids and certain minerals. Importantly, ultrasound alone does not uniformly alter all measured endpoints; rather, its effect is most evident when combined with the temperature conditions that favour enzyme expression or activity, indicating synergistic rather than purely additive interactions.

The divergent outcomes across antioxidant assays, together with the compositional shifts induced by simulated gastrointestinal digestion, reflect the biochemical heterogeneity and differential stability of the released metabolites. In particular, the USC‑36 treatment yielded the greatest enhancement in flavonoid bioaccessibility and FRAP activity, whereas total phenolic content declined in the 36 °C groups and CUPRAC values remained largely unresponsive. These contrasting outcomes likely reflect several concurrent processes: (i) selective degradation or transformation of certain phenolic fractions during acidic gastric and enzymatic intestinal phases [32]; (ii) conversion of glycosylated precursors into aglycones that are measured differently by colorimetric assays [33]; and (iii) because FRAP measures ferric‑reducing capacity while CUPRAC evaluates cupric ion reduction, digestion‑derived metabolites can yield divergent responses depending on their redox potential, structural features, and molecular size, highlighting the biochemical heterogeneity of the released fractions. These electron transfer-based assays exhibit different selectivity toward antioxidant compounds, with CUPRAC providing more additive results in complex mixtures compared to FRAP, which can show surprising overoxidations and large deviations from additivity when testing phenol-thiol combinations [34]. Thus, variations in total phenolics and flavonoids, even when not captured by a single assay, can influence biological antioxidant potential through their distinct redox properties, structural interactions, and digestive stability.

Mineral bioaccessibility is element-specific and shaped by processing interactions. The USC-36 maximizes calcium and iron release during digestion, whereas magnesium bioaccessibility is higher in 30 °C treatments. These patterns can be explained mechanistically as fermentation facilitates phytate breakdown and loosens the macromolecular matrix to improve mineral solubility [35], whereas ultrasound cavitation enhances cellular permeability and exposes mineral‑binding sites, thereby promoting Ca^2+^ uptake and redistribution [36]. Conversely, polyphenol–mineral interactions can selectively chelate divalent cations, with magnesium particularly prone to sequestration, thereby reducing its bioaccessibility despite matrix disruption [9]. Compound‑specific effects, such as the low bioaccessibility of quercetin and the inhibitory impact of catechin on protein digestibility, illustrate how phenolic structures influence nutrient release [37]. Processing interventions like ultrasound and fermentation may counteract these barriers by disrupting the matrix and weakening binding forces, though mineral leaching remains a risk [29]. In line with our findings, the positive association between flavonoids and calcium, together with the strong TFC–FRAP correlation, suggests that flavonoid–mineral interactions contribute to both mineral solubility and antioxidant responses, while microbiota‑mediated transformations may further shape these outcomes [38].

Correlation analysis provided further insight into the digestion-dependent transformations occurring across treatments. Following simulated gastrointestinal digestion, TFC showed strong positive correlations with FRAP, CUPRAC, and iron, indicating that flavonoid release was a primary contributor to the enhanced antioxidant capacity and improved iron bioaccessibility. The strongest positive relationship was observed between TFC and calcium, suggesting that the same structural modifications facilitating flavonoid release, such as matrix softening and partial cell-wall disruption, also promoted calcium solubilization. FRAP demonstrated additional positive correlations with calcium and iron, reinforcing the mechanistic connection between antioxidant-related metabolites and mineral release after digestion.

In contrast, both TFC and FRAP exhibited negative correlations with magnesium, reflecting distinct release dynamics for this mineral compared with calcium and iron. These opposing relationships indicate that the gastrointestinal conditions and processing parameters that favor phenolic and mineral release do not uniformly enhance magnesium solubility. Overall, the emergence of these digestion-dependent correlations supports the conclusion that ultrasound pretreatment combined with elevated fermentation temperature enhances specific structural and biochemical pathways governing the bioaccessibility of flavonoids and key minerals.

This study provides novel insights by integrating ultrasound pretreatment with controlled fermentation temperatures and assessing their combined effects on the simulated gastrointestinal bioaccessibility of phenolics, flavonoids, and minerals in tempeh. The simultaneous evaluation of multiple compound classes and antioxidant responses strengthens the comprehensiveness of the findings and highlights the potential of processing strategies to modulate nutrient release. However, several limitations must be acknowledged: the *in vitro* gastrointestinal model cannot fully replicate intestinal absorption, systemic metabolism, or host–microbiome interactions that ultimately determine bioavailability. Moreover, sensory quality, consumer acceptability, and storage stability were not addressed, which are essential for translation into food applications. Although TPC and TFC were evaluated using established spectrophotometric assays, these methods do not quantify individual phenolic or flavonoid compounds. Chromatographic approaches such as HPLC or LC–MS/MS were not included in this study and should be incorporated in future work to characterize specific bioactive constituents and further elucidate how ultrasound pretreatment and fermentation temperature influence their release and bioaccessibility. Taken together, these strengths and limitations underscore both the novelty and the boundaries of the present work, while pointing to the need for compound‑level profiling, *in vivo* validation, and consumer‑oriented assessments to fully establish the nutritional and technological relevance of ultrasound pretreatment fermentation in tempeh production.

The present findings open important avenues for advancing the role of tempeh as a functional food within sustainable dietary strategies. By demonstrating that ultrasound processing and fermentation temperature can enhance the bioaccessibility of phytonutrients, including flavonoids and minerals, this work provides a mechanistic basis for optimizing plant‑based foods to address nutrient needs in vulnerable populations. More broadly, improving the release and stability of bioactive compounds from legumes aligns with global efforts to promote sustainable protein sources that deliver both nutritional adequacy and chronic disease risk reduction. Future research should therefore extend these *in vitro* observations to *in vivo* models and clinical trials, with a focus on the aging population and postmenopausal women, while also integrating sensory evaluation, consumer acceptance, and life‑cycle sustainability assessments. Such work will be critical to establish ultrasound pretreatment combined with controlled fermentation not only as a technological innovation but also as a strategy to support healthy aging and environmentally responsible diets.

## 5 Conclusion

This study demonstrates that combining ultrasound pretreatment with elevated fermentation temperatures enhances the release and gastrointestinal bioaccessibility of phytoactive compounds and essential minerals in tempeh. Ultrasound pretreatment promoted disruption of the soybean matrix, facilitating the release of bound polyphenols and minerals, while higher fermentation temperatures stimulated microbial enzymatic activity and the formation of bioactive metabolites. Collectively, these mechanistic effects led to significant improvements in flavonoid, calcium, and iron bioaccessibility under simulated digestive conditions.

From an applied perspective, these findings suggest that integrating ultrasound pretreatment during soaking or cooking with controlled fermentation temperatures represents a promising strategy for producing nutritionally enhanced fermented soybean products. Nevertheless, further research is needed to assess the scalability of ultrasound pretreatment, its energy requirements, and cost-effectiveness in industrial applications. Additionally, *in vivo* studies and sensory evaluations would be valuable to confirm the nutritional benefits, consumer acceptance, and long-term functional impact of these preprocessing methods. Such efforts will facilitate the translation of current findings into practical applications for the food industry and support the development of functional plant-based foods with improved health-promoting potential.

## Funding statement

This study was funded by the German Academic Exchange Service (DAAD), with Iskandar Azmy Harahap as the grant holder. The publication of this article was funded by the Open Access fund of Leibniz Universität Hannover.

## CRediT authorship contribution statement

**Iskandar Azmy Harahap:** Writing – review & editing, Writing – original draft, Visualization, Validation, Software, Resources, Project administration, Methodology, Investigation, Funding acquisition, Formal analysis, Data curation, Conceptualization. **Joanna Suliburska:** Writing – review & editing, Supervision, Methodology. **Daniela Weber:** Writing – review & editing. **Tuba Esatbeyoglu:** Writing – review & editing, Supervision, Funding acquisition.

## Declaration of competing interest

The authors declare that they have no known competing financial interests or personal relationships that could have appeared to influence the work reported in this paper.
